# The Effect of Coatings and Nerve Growth Factor on Attachment and Differentiation of Pheochromocytoma Cells

**DOI:** 10.3390/ma11010060

**Published:** 2017-12-31

**Authors:** Anna Orlowska, Pallale Tharushi Perera, Mohammad Al Kobaisi, Andre Dias, Huu Khuong Duy Nguyen, Shahram Ghanaati, Vladimir Baulin, Russell J. Crawford, Elena P. Ivanova

**Affiliations:** 1 Frankfurt Orofacial Regenerative Medicine, University Hospital Frankfurt, Theodor-Stern-Kai 7, D-60590 Frankfurt am Main, Germany; a.b.orlowska@gmail.com (A.O.); andreecdias@gmail.com (A.D.); s.ghanaati@med.uni-frankfurt.de (S.G.); 2 Departament d’Enginyeria Quimica, Universitat Rovira i Virgili, 26 Avenue dels Paisos Catalans, 43007 Tarragona, Spain; va.baulin@gmail.com; 3 Department of Chemistry and Biotechnology, School of Science, Swinburne University of Technology, P.O. Box 218, Hawthorn VIC 3122, Australia; pgperera@swin.edu.au (P.T.P.); malkobaisi@swin.edu.au (M.A.K.); huukhuongduynguyen@swin.edu.au (H.K.D.N.); 4 School of Science, RMIT University, P.O. Box 2476, Melbourne VIC 3001, Australia; Russell.Crawford@rmit.edu.au

**Keywords:** nerve growth factor (NGF), poly-L-lysine, fibronectin, laminin, PC12 neuronal differentiation

## Abstract

Cellular attachment plays a vital role in the differentiation of pheochromocytoma (PC12) cells. PC12 cells are noradrenergic clonal cells isolated from the adrenal medulla of *Rattus norvegicus* and studied extensively as they have the ability to differentiate into sympathetic neuron-like cells. The effect of several experimental parameters including (i) the concentration of nerve growth factor (NGF); (ii) substratum coatings, such as poly-L-lysine (PLL), fibronectin (Fn), and laminin (Lam); and (iii) double coatings composed of PLL/Lam and PLL/Fn on the differentiation process of PC12 cells were studied. Cell morphology was visualised using brightfield phase contrast microscopy, cellular metabolism and proliferation were quantified using a 3-(4,5-dimethylthiazol-2-yl)-5-(3-carboxymethoxyphenyl)-2-(4-sulfophenyl)-2H-tetrazolium (MTS) assay, and the neurite outgrowth and axonal generation of the PC12 cells were evaluated using wide field fluorescence microscopy. It was found that double coatings of PLL/Lam and PLL/Fn supported robust adhesion and a two-fold enhanced neurite outgrowth of PC12 cells when treated with 100 ng/mL of NGF while exhibiting stable metabolic activity, leading to the accelerated generation of axons.

## 1. Introduction

The pheochromocytoma (PC12) cell line is commonly used in in vitro studies to examine neuronal differentiation and neurotoxicity implicated in neurodegenerative disease [1,2]. PC12 cells are noradrenergic clonal cells originating from *Rattus norvegicus* transplantable pheochromocytoma [1]. They exhibit a reversible response to nerve growth factor (NGF). After NGF exposure, PC12 cells acquire characteristic phenotypic properties associated with sympathetic neuron-like cells, which includes the inhibition of proliferation, outgrowth of neurites, and the possibility of being electrically excitable [1,2,3]. Upon differentiation, the neuron-like PC12 cells start to express various integral proteins that are responsible for neurite growth [1] and can transmit signals along the axons [4,5].

In laboratory conditions, PC12 cells attach poorly to polystyrene (PS) tissue culture surfaces [4,6] where they grow mostly as floating cell aggregates [6]. This poor adhesion ultimately results in insufficient levels of neurite outgrowth [4]. Functionalization of the surface can improve the attachment of the PC12 cells. Previously, it was shown that the pre-treatment of the substratum surface with proteins, especially the extracellular matrix components, enhanced not only cell adhesion, but also their growth, morphology, migration, and differentiation, increasing their life-span [7].

Various substratum coatings used in the formation of PC12 neuronal processes have been reported, e.g., glycoproteins, collagen (type IV from human placenta, type I) [8,9,10,11], laminin (Lam) [12], and fibronectin (Fn) [3]. In addition, it was shown that polyornithine [3,13,14,15,16], poly-D-lysine, and poly-L-lysine (PLL) can be used to enhance the attachment of PC12 cells [8,9,10,11]. Although Tomaselli et al. [17] have shown that Lam and collagen type IV coated surfaces promoted PC12 adhesion to a greater degree than Fn, no systematic assessment of the suitability of various coatings that would enhance the attachment and differentiation of PC12 cells has been performed. Therefore, the aim of our study was to investigate the effect of five coating types, including Lam, Fn, and PLL (and various combinations of these coatings) on the attachment, proliferation, and differentiation of PC12 cells. The extent of differentiation of the PC12 cells using different coatings was monitored by measuring a set of parameters related to cellular functions as a function of NGF concentration. The proliferation and metabolic activity of the PC12 cells were analysed using an MTS (3-(4,5-dimethylthiazol-2-yl)-5-(3-carboxymethoxyphenyl)-2-(4-sulfophenyl)-2H-tetrazolium) assay. The morphology and neuron-like characteristics of the cells were analysed by using brightfield phase contrast microscopy and wide field fluorescence microscopy.

## 2. Materials and Methods

### 2.1. PC12 Cell Line

Commercially-available PC12 CRL-1721™ cells were obtained from the American Type Culture Collection (American Type Culture Collection (ATCC), Manassas, VA, USA). The cells were cultured in 75 mm^2^ tissue culture flasks with complete Gibco™ 1640 RPMI medium supplemented with 10% horse serum (HS, Thermo Fisher Scientific Australia Pty Ltd., Melbourne, Australia), 5% fetal bovine serum (FBS, Thermo Fisher Scientific Australia Pty Ltd., Melbourne, Australia) and 1% Penicillin/Streptomycin (Pen/Strep, Thermo Fisher Scientific Australia Pty Ltd., Melbourne, Australia). The cells were maintained according to standard protocols [4,18] at 37 °C in a 95% humidified incubator with 5% CO_2_. The medium was changed every 2 days, and passaged accordingly when the confluence reached 90%. 

### 2.2. Coatings

#### 2.2.1. Poly-L-lysine

Sterile-filtered poly-L-lysine (PLL) (molecular weight 150,000–300,000 Da) was purchased from Sigma-Aldrich (Sydney, Australia) and used at the recommended concentration of 0.01% *w*/*v* in water. The sterile filtered solution was stored at 4 °C until required.

#### 2.2.2. Fibronectin

Fibronectin (Fn) was purchased from Sigma-Aldrich (Sydney, Australia) in lyophilized powder form. An aqueous working solution of 40 µg/mL concentration was prepared and stored at −20 °C until required.

#### 2.2.3. Laminin

Laminin (Lam) derived from a mouse Engelbreth-Holm-Swarm (EHS) sarcoma was purchased from Sigma-Aldrich (Sydney, Australia). An aqueous working solution of 10 µg/mL concentration was prepared and stored at −20 °C until required.

### 2.3. Atomic Force Microscopy

The surface topography of the various substrata was visualized using an Innova^®^ atomic force microscope (Bruker, Billerica, MA, USA). A phosphorous-doped silicon probe (MPP-31120-10, Bruker, Billerica, MA, USA) was employed in tapping mode for all measurements performed in air, at a temperature of approximately 22 °C. The atomic force microscopy (AFM, Veeco, Bruker, Billerica, MA, USA) scans, obtained over a 10 µm × 10 µm surface area with 512 × 512 data points, were processed using Gwyddion software (Version 2.49, Czech Metrology Institute, Brno, Czech) [19]. Analysis was conducted using three AFM micrographs for each sample, and for two samples of each substratum surface. The surface roughness parameters derived from the AFM data included the arithmetic average height (*S*_a_), root mean square deviation from the mean plane (*S*_q_), maximum height of the profile (*S*_max_), skewness (*S*_skw_), and kurtosis (*S*_kur_), as described in detail elsewhere [20].

### 2.4. Differentiation of PC12 Cells

PC12 cells were seeded onto 24-well polystyrene (PS) tissue culture plates at a density of 3 × 10^4^ cells/mL in Gibco™ 1640 Roswell Park Memorial Institute (RPMI) medium (1% HS, 1% FBS and 1% Pen/Strep) supplemented with 0, 25, 50, and 100 ng/mL Nerve Growth Factor (NGF) (mouse recombinant NGF 7S, Sigma-Aldrich, Sydney, Australia). Prior to seeding, the tissue culture dish (Sarstedt AG & Co., Nümbrecht, Germany) was prepared with different coatings, with each being prepared in triplicate. Non-coated plates were employed as control surfaces. PLL was added to cover the entire surface area and incubated at 25 °C for 2 h. The wells were washed with phosphate-buffered saline (PBS) and a second coating of Fn or lam was added and incubated at 25 °C for 2 h. The non-coated wells of the tissue culture dishes were used as control surfaces. At least two technical replicates were completed. The culture medium on the samples was changed every second day.

### 2.5. Metabolic Activity

The metabolic activity of the PC12 cells was determined using the CellTiter 96^®^AQueous One Solution Cell Proliferation Assay (Promega, Sydney, Australia). The assay was performed by adding the tetrazolium compound to the PC12 cell culture at a 10% ratio of the final volume. This allowed the reduction of (3-(4,5-dimethylthiazol-2-yl)-5-(3-carboxymethoxyphenyl)-2-(4-sulfophenyl)-2H-tetrazolium) (MTS) to formazan, resulting in the formation of a coloured precipitate. The absorbance of the resultant solution was recorded at a wavelength of 490 nm after incubation for 90 min at 37 °C using a FLUOstar Omega microplate reader (BMG LABTECH, Cary, NC, USA).

### 2.6. Assessment of Neurite Outgrowth

A neurite outgrowth analysis was performed using representative digitized photomicrographs of each well, obtained using a phase contrast brightfield inverted Olympus microscope (CKX41, Olympus, Tokyo, Japan) equipped with a Panasonic camera (DMC-GH3). The quantity of differentiated cells was established by the visual detection of neurites. Evaluation of neurite growth was carried out by manually tracking the length of the neurites on each cell using NeuronJ software (ImageJ plugin; NIH, Bethesda, MD, USA). This procedure was conducted for all cells in a field where the entire neurite could be visualized.

### 2.7. Widefield Fluorescence Microscopy

After a 5-day incubation period, the cells were washed with PBS and fixed with 4% paraformaldehyde for 15 min. After fixation, the cells were washed with PBS and cell membranes were stained using Wheat Germ Agglutinin, Alexa Flour^™^ 488 (WGA, Thermo Fisher Scientific Australia Pty Ltd., Melbourne, Australia). The nucleus was stained using DAPI (4’,6-diamidino-2-phenylindole). Samples were then imaged using a Nikon Eclipse Ti-E epifluorescence inverted microscope (Nikon Instruments Inc., Tokyo, Japan). Sequential images were acquired using DAPI and GFP-B standard series filter cubes and analysed using FIJI (ImageJ) software (k.1.45, National Institute of Mental Health, Bethesda, MD, USA) [21].

### 2.8. Statistical Analysis

Statistically significant differences (*p* < 0.05, *p* < 0.01, *p* < 0.001, *p* < 0.0001) were calculated among the various groups using a two-way ANOVA analysis followed by Tukey’s multiple comparison test. All statistical analyses were carried out using the GraphPad Prism 7 statistical software package (GraphPad Software, Inc., San Diego, CA, USA).

## 3. Results

Single- and two-component coatings were investigated in this study. The glycoproteins, such as Lam and Fn, were selected because they are typical components of the extracellular matrix [5,17,22,23]. It has been reported that Lam and Fn positively influenced the outgrowth of neurons, axonal guidance, differentiation and cell proliferation [5,22]. Since PLL was reported to facilitate cell attachment and improve the differentiation of PC12 cells [24], PLL was also selected for analysis. In addition to single component coatings, PLL/Lam and PLL/Fn combination coatings were also studied.

### 3.1. Protein Distribution on the Substratum

**Figure 1:**
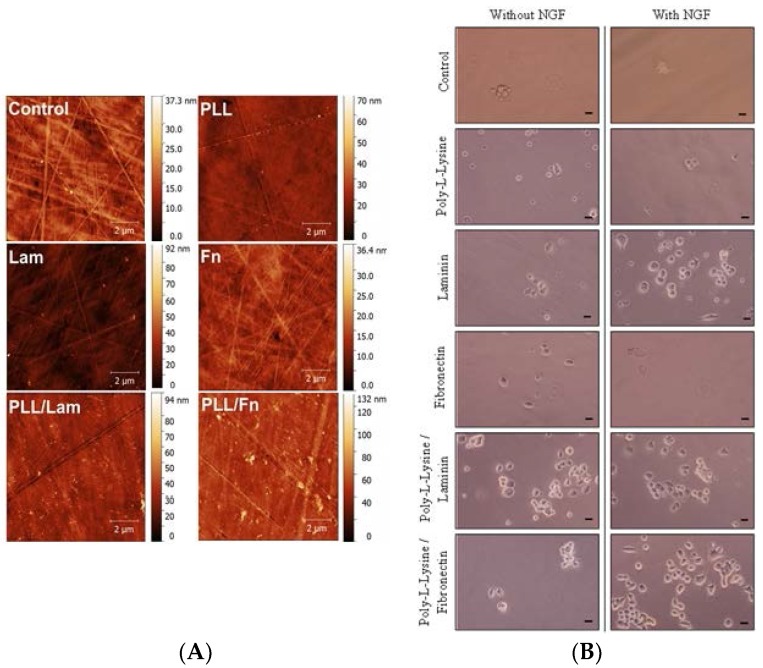
(**A**) AFM analysis of surfaces with different coatings. The AFM micrographs show an even distribution of single and two-component coatings on the surfaces of the polystyrene substratum. (**B**) Attachment and initial differentiation of PC12 cells after two days of incubation in the presence and absence of NGF solution. Cells were seeded at a density of 3 × 10^4^ cells/mL on poly-L-lysine, laminin, fibronectin, poly-L-lysine/laminin, and poly-L-lysine/fibronectin-coated wells. The cells were grown in a medium that was supplemented with human recombinant NGF (50 ng/mL). The control wells did not contain any coating. In these experiments the PC12 cells appeared to attach to the surface in clusters. Scale bar is 5 µm.

An AFM analysis confirmed that an even distribution of the coatings was present on the plastic surfaces of the cell culture wells (Figure 1A). The *S*_a_ of the control (2.2 ± 0.2 nm) was found to be similar to that present on the substrata with a single coating, namely, Fn (1.8 ± 0.1 nm) and PLL (2.1 ± 0.7 nm). The Lam coating exhibited a higher *S*_a_ of 2.9 ± 0.4 nm. The PLL/Fn and PLL/Lam dual component coatings were found to have *S*_a_ of 3.4 ± 0.4 nm and 5.8 ± 1.2 nm, respectively.

### 3.2. PC12 Cell Attachment and Initial Differentiation in the Presence of NGF

**Figure 2:**
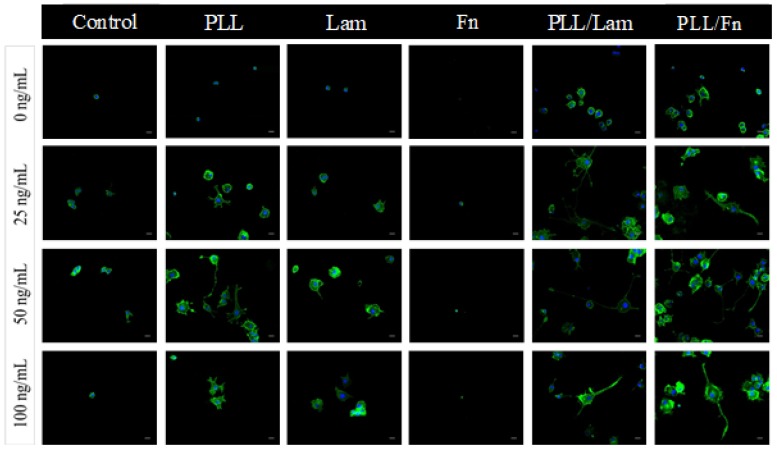
Comparative differentiation of PC12 cells on substrata with single- and two-component coatings in the presence of NGF solution. The NGF concentrations used were 0, 25, 50, and 100 ng/mL. Cells were grown over a period of five days; fixed in 4% PFA for 15 min and stained with WGA-488 (membrane, green), DAPI (nuclei, blue). Significant neurite outgrowth was observed for cells grown on substrata with double coatings containing poly-L-lysine/laminin and poly-L-lysine/fibronectin. Substrata containing single coatings of poly-L-lysine and laminin exhibited poor cell differentiation.

The extent of growth of the PC12 cells on substrata containing the five different coating types, together with various concentrations of NGF, was investigated in order to identify the most suitable growth conditions for the stimulated attachment and differentiation of PC12 cells into neuron-like cells. Cell attachment, growth, and differentiation patterns were monitored over a five-day period (Figure 1B and Figure 2). The initial differentiation and attachment propensity of the PC12 cells after 48 h (day 2) was studied using phase-contrast brightfield microscopy (Figure 1B).

The densities of attached cells on the substrata possessing a single Lam coating and dual-component coatings were found to be comparable, whereas isolated, spherically-shaped cells were observed without protrusions on substrata containing the PLL and Fn coatings. Enhanced levels of cell attachment were detected on substrata containing the PLL/Fn and Lam coatings in the presence of 50 ng/mL NGF (Figure 1B). In the absence of 50 ng/mL NGF, only a few cells were observed to have attached to these surfaces. These results confirmed that the presence of a coating is essential for robust cell attachment to be supported, whereas it was apparent that NGF could act as a co-factor for achieving enhanced levels of cell attachment.

In order to compare and confirm the cell differentiation processes on the various substrata being investigated, PC12 cells were stained with a membrane specific protein and imaged using wide field fluorescence microscopy (Figure 2). Analysis of the fluorescence micrographs indicated that the dual component PLL/Fn and PLL/Lam coatings supported enhanced levels of PC12 cell differentiation, as evidenced by the presence of neurite outgrowth. In contrast, on PLL- and Lam-coated surfaces, the PC12 cells exhibited a lower degree of differentiation, with no or just a few non-differentiated cells being observed on the Fn- and non-coated surfaces.

PC12 cells incubated on Fn- or non-coated surfaces exhibited little to no attachment propensity in both the presence and absence of NGF. These results are consistent with previously reported data that suggested that NGF acted as a co-factor in enhancing the attachment and differentiation of cells, however, the surface coating itself was found to be the main factor determining the extent to which cells would attach to surfaces.

### 3.3. PC12 Cells Metabolic Activity and Proliferation

**Figure 3:**
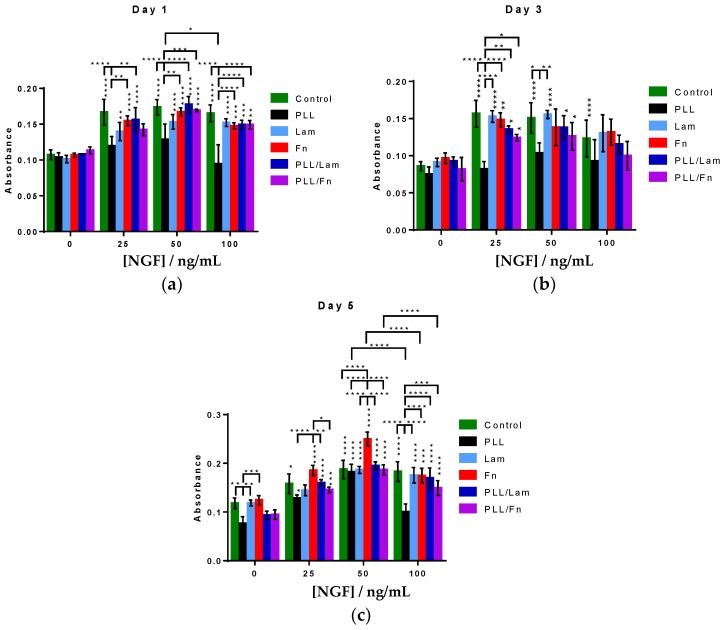
Comparative analysis of the proliferation of PC12 cells on substrata with different coatings in the presence of NGF solution. PC12 cells were grown over a period of 5 days in the presence of 0, 25, 50, or 100 ng/mL NGF. Incubation of PC12 cells on substratum surfaces in the presence of 50 ng/mL NGF resulted in an increased amount of attachment on day (**a**) 1, (**b**) 3 and (**c**) 5 compared to the other NGF solutions. Results are presented as the mean ± standard deviation. Unless otherwise specified, statistically significant differences in cell proliferation grown on the different substrata are shown versus the absence of NGF solution. “*” indicates the degree of statistically significant differences.

The differentiation of PC12 cells into neuron-like cells has been found to be accompanied by an arrest in the post-mitotic G0 stage in the cell cycle, which in turn decreased their potential to proliferate [1,2,3,25]. A commonly used method for the evaluation of proliferation activity is through monitoring cell metabolic activity via an MTS assay [26]. In this work, an MTS assay was employed to study the metabolic activity of the PC12 cells while attached to the different substratum surfaces. PC12 cells grown on PLL-coated substrata exhibited an increased growth after five days when both 25 and 50 ng/mL NGF was present, compared to growth in the absence of NGF (Figure 3). At a concentration of 100 ng/mL NGF, however, a decrease in cell growth was observed. This decrease is likely to be associated with a progression of the cells into an arrested G0 stage, which is characteristic of PC12 cells that have undergone a differentiation to form neuron-like cells. Analysis of the metabolic activity of the PC12 cells 24 h after seeding (day 1) indicated that the metabolic activity of the cells increased in the presence of NGF, however, when cells were incubated on a PLL-coated substratum, an increase in metabolic activity was not observed. After the third day of growth, a similar metabolic activity was observed for all cells attached to all surfaces.

After the 5th day, cell proliferation was found to increase with increasing NGF concentration for all substrata. Cells incubated in the presence of substrata coated with PLL, Fn and PLL/Fn, and 100 ng/mL NGF exhibited a statistically significant decrease in proliferation compared to that observed in the presence of 50 ng/mL NGF.

### 3.4. Neurite Outgrowth

**Figure 4:**
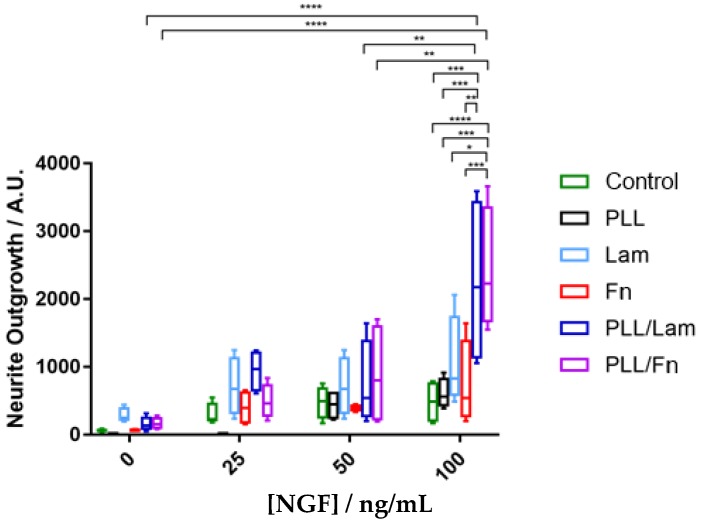
Quantification of neurite extension. The PC12 cells were grown on various coatings over a period of five days and in the presence of 0, 25, 50, and 100 ng/mL NGF. Over fifty fields of view were analysed for each condition. The results indicated a two-fold greater neurite outgrowth occurring on substrata containing the dual-component coatings of poly-L-lysine/laminin and poly-L-lysine/fibronectin with increasing NGF concentrations. Results are presented as minimum, 1st quartile, median, 3rd quartile, and maximum. “*” indicates the degree of statistically significant differences.

The neurite outgrowth was evaluated for individual cells, grown under different experimental conditions. The results are presented in Figure 4. A comparative analysis of these data indicated that increases in NGF concentration stimulated the outgrowth of neurites, despite the metabolic activity of the cells being greater at 50 ng/mL (Figure 3). It was also found that substrata with two-component coatings stimulated the outgrowth of neurites in the presence of a 100 ng/mL NGF solution (Figure 4). PC12 cells incubated in the presence of substrata with or without a single coating showed reduced levels or no outgrowth of neurites, most likely because of low cell attachment occurring on these surfaces. The substrata possessing dual component coatings stimulated the differentiation of neurites (Figure 4) after cell proliferation had been arrested (in the presence of 100 ng/mL NGF) after the 5th day of incubation (Figure 3).

## 4. Discussion

**Figure 5:**
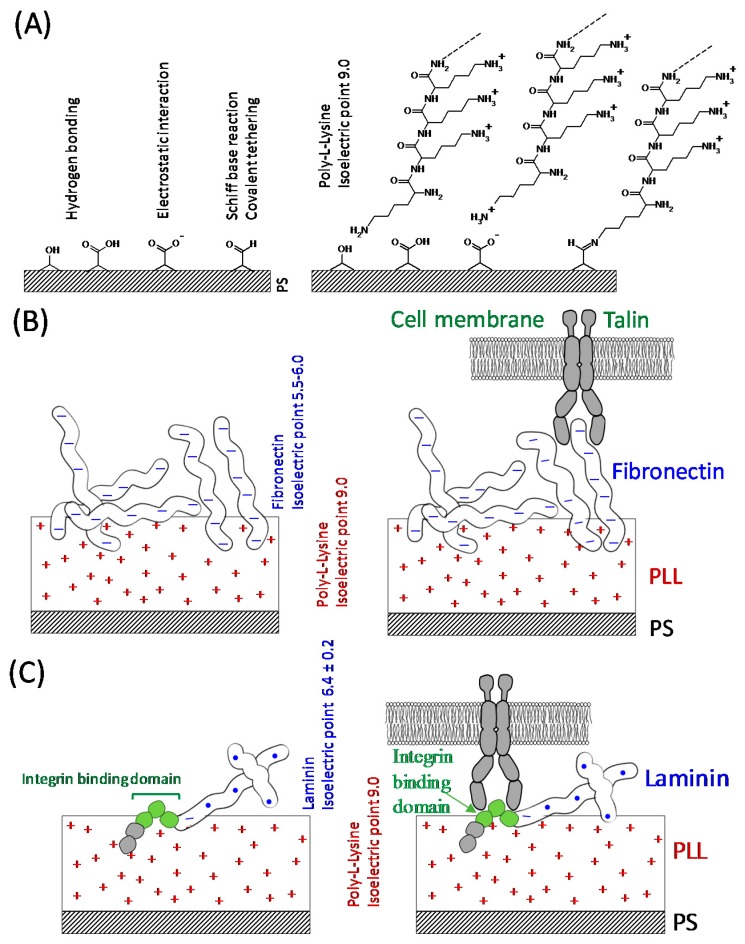
Schematic interpretation of the bio-interfaces of the PC12 cells undergoing attachment onto the substratum samples. (**A**) Possible chemical interactions between the polystyrene substratum and the coating materials. The aldehyde and ketone groups present on the surface of polystyrene undergo a Schiff base reaction and bind covalently with the amine groups. (**B**) negatively-charged fibronectin (pI 5.5–6.0) binding to the positively charged poly-L-lysine coating via electrostatic interactions. (**C**) laminin (pI 6.4) with a net negative charge also binds electrostatically to the poly-L-lysine layer. Laminin interacts with the PC12 cell surface receptors via the integrin binding domain.

The results of this study have demonstrated that PC12 cells incubated in the presence of substrata with dual-component coatings composed of PLL/Lam or PLL/Fn exhibited enhanced attachment, proliferation, and outgrowth of neurites. It is likely that this enhancement has occurred because PLL offers an increased number of cationic sites that can bind to the ionic sites present on the polystyrene surfaces of the tissue culture wells via hydrogen bonding or electrostatic interactions (Figure 5A). The glycoproteins, while effectively binding to PLL via electrostatic interactions (Fn and Lam isoelectric point of 5.5–6.0 [27] and 6.4 [28], respectively (Figure 5B,C)), also provided specific binding sites for the PC12 cells, allowing greater levels of cell attachment to take place [29], because cell surface receptors that promote cellular adhesion to extracellular matrices [30] are known to facilitate adherence the PC12 cells [30]. It was also demonstrated that the cell attachment domain of Fn consists of potential chains of β-turns, which form the most hydrophilic region for cell attachment [29]. The integrin binding domain present on Lam [27] can bind with the integrin receptors present on the surface of PC12 cells (Figure 5C), resulting in adhesion and the initiation of cell differentiation. The PC12 cells strongly attached to the substrata containing a PLL coating due to the negatively-charged cell membrane and nonspecific electrostatic interactions with the highly positively-charged PLL surface (Figure 5A) [24]. The single-component coatings composed of only glycoproteins were most likely not able to support a robust attachment onto the surfaces of the tissue culture wells because of the weak binding affinity of this protein to the well surface. As a result, the PC12 cells would not have been able to withstand the washing procedures taking place during the incubation period.

## 5. Conclusions

In summary, substrata containing the two-component coatings of PLL/Lam and PLL/Fn in the presence of 100 ng/mL NGF solution were found to result in the greatest levels of attachment of PC12 cells followed by early stimulation of cell differentiation and neurite outgrowth.
